# Genotoxic effects of X-rays in buccal mucosal cells in children subjected to dental radiographs

**DOI:** 10.1038/bdjopen.2016.1

**Published:** 2016-03-25

**Authors:** Naveena Preethi, Nagarathna Chikkanarasaiah, Shakuntala S Bethur

**Affiliations:** 1 Department of Pedodontics and Preventive Dentistry, Rajarajeswari Dental College and Hospital, Bangalore, India

## Abstract

**Objectives/Aims:**

Bitewing and digital dental panoramic radiographs have become important adjuvants for successful dental practice in pediatric dentistry. Both methods lead to genetic changes in the oral buccal epithelium that have not yet been satisfactorily explored. The aim of the present study was to evaluate the genotoxic effects induced by X-ray radiation from bitewing and panoramic dental radiography in exfoliated buccal epithelial cells of children, using the Buccal Micronucleus Cytome assay.

**Materials and Methods:**

Children (*n*=40) who met the inclusion criteria and provided signed informed consent were included in the study. Children were selected for undergoing bitewing radiographs (group 1; *n*=20) or digital dental panoramic radiographs (group 2; *n*=20). Exfoliated buccal mucosal cells were obtained by scraping the right/left buccal mucosa with a wooden spatula immediately before the X-ray exposure and 10±2 days after exposure.

**Results:**

The frequency of micronuclei increases significantly post exposure to both bitewing and digital dental panoramic radiography in children, but the frequency was higher in bitewing radiographs.

**Conclusion:**

It was concluded that the frequency of micronuclei increases post exposure to both bitewing and digital panoramic radiographs. Increased radiation exposure results in an increase in micronuclei frequency.

## Introduction

Roentgenography is an important diagnostic method with wide application in pediatric practice. There is a tremendous need for roentgenography in children compared with adults as there is a greater concern with regard to growth and development in children, and factors that alter them.^1^

Over the years, roentgenography has become an important adjuvant to successful dental practice, especially in pediatric dentistry. Primarily, a bitewing radiograph, which is also known as an interproximal intraoral radiograph, was used. Adjuvant to this, the panoramic radiograph, also known as rotational panoramic imaging, an extra oral radiographic technique that images both the maxilla and the mandible on single exposure, is also used. This is mostly indicated for the assessment of growth and development of jaws of children and adolescents, to view the mixed dentition stages, and the status and stages of resorption of primary dentition. In both radiographic methods, the oral buccal epithelium is directly exposed to ionizing X-ray radiation.^2^

Even though X-rays are widely used for diagnostic and therapeutic reasons^3^, there is considerable concern with regard to the potential harmful effects associated with radiation exposure as there is no safe margin of dosage. Ionizing radiation either acts directly on the DNA molecule or indirectly through the formation of reactive compounds that interact with the DNA molecule resulting in cytotoxicity of the cell.^4^

Children may be vulnerable to greater risk for genetic damage from radiation exposure compared with adults, as young, and rapidly growing tissues are more radiosenstive than mature tissue. Further, the intercellular effects of ionizing radiation are cumulative and may likely lead to the development of radiation-induced tumors. Nowadays, various biomarkers are used to assess DNA-induced genetic damages. One such reliable biomarker is The Buccal Micronucleus Cytome (BMCyt) assay, which detects genetic damage with the presence of micronucleus (MN).

The BMCyt assay is a suitable internal dosimeter for revealing tissue-specific genotoxic damage in individuals exposed to carcinogens.^5^ The analysis of micronuclei has gained popularity as a biomonitoring assay for human genotoxic exposure and its effects because it is noninvasive, the scoring is simple, it requires shorter training, it is less time-consuming,^6^ and precision is obtained from scoring a large number of cells with better patient acceptance.^7^ It is also a sensitive short-term assay for the detection of genotoxicants.^8^

The possible genotoxic effects from bitewing and panoramic dental radiography as assessed by MN occurrence have not yet been satisfactorily explored, as it has been investigated in only a few studies on exfoliated cells. Hence, the aim of the present study was to evaluate the genotoxic effects induced by X-ray radiation from bitewing and panoramic dental radiography in exfoliated buccal epithelial cells collected from children who were subjected to routine diagnostic procedures, using the BMCyt assay.

## Materials and methods

### Study design

The present study was conducted after obtaining approval from the Instutional Ethical Committee and signed written informed consent from parents of 40 healthy children, who were advised to undergo bitewing or digital dental panoramic dental radiographs as a part of their diagnostic procedure and who were referred to the Department of Pedodontics and Preventive Dentistry, Rajarajeswari Dental College and Hospital, Bangalore.

### Inclusion criteria

1. Healthy children subjected to bitewing and digital dental panoramic radiographs for various diagnostic purposes.
2. Age between 6 and 12 years.
3. No exposure to head and neck radiation before the study.

### Exclusion criteria

1. Presence of systemic diseases or being differently abled.
2. Radiographic exposure within the last 6 months.

### Screening and sample collection

In this *ex vivo* study, 40 children who met the inclusion criteria were asked to rinse their mouth thoroughly with normal water to remove any unwanted debris before sample collection. Twenty children were selected for bitewing radiographs (group 1) and 20 children for digital dental panoramic radiographs (group 2). Exfoliated buccal mucosal cells were obtained by scraping the right/left buccal mucosa with a wooden spatula immediately before the X-ray exposure and 10±2 days after exposure.

### Exfoliated buccal cell staining for microscopy

The microscope slides with fixed cells were coded according to group and subject. Each slide was immersed for 1 min in two Coplin jars containing 50% (vol/vol) and 20% (vol/vol) ethanol, respectively. The cells were washed for 2 min in a Coplin jar containing Milli-Q water. The slides were then transferred to a Coplin jar containing 5 M Hcl for 30 min and rinsed in running tap water for 3 min. They were then drained, but not allowed to dry out, and were placed in a Coplin jar containing Schiff’s reagent for 60 min in the dark at room temperature. The slides were then rinsed in running tap water for 5 min and rinsed well in Milli-Q water. The cells were counterstained by immersing in a Coplin jar containing 0.2% (wt/vol) Light Green for 20–30 s and rinsed well in Milli-Q water. To blot away any residual moisture, the slides were immediately placed face down onto Dr Watts no. 1 filter paper. They were then placed on a slide tray and allowed to dry for ~10–15 min. The efficiency of staining and the density of cells were examined at ×100 and ×400 magnification, respectively. Thereafter, the slides were dried completely for at least 30 min before placing a coverslip with distrene dibutylphthalate xylene (DPX) and observed using transmitted light microscopy. The nuclei and micronuclei were magenta in color, whereas the cytoplasm appeared pale blue/green. The cells were viewed under fluorescence with a far-red filter, because Feulgen-stained DNA appears bright red in color under these conditions.

### BMCyt assay analysis

Coded slides were examined for 1,000 cells per subject at ×400 magnification. In each slide, 250 intact epithelial cells were scored for the presence of micronuclei. As four slides per subject were scored, a total of 1,000 cells were scored per subject. Abnormalities were identified by fluorescence microscopy. Slides were evaluated using the criteria of Tolbert *et al.*^8^.

### Statistical method

The data obtained for both groups, i.e., for group 1 before exposure and after exposure and for group 2 before exposure and after exposure, were tabulated and subjected to statistical analysis with SPSS software version 20.0 using the Mann–Whitney test. *P* values <0.05 were considered statistically significant.

## Results

From this *ex vivo* study conducted to evaluate the extent of genetic changes on the basis of MN frequency in exfoliated buccal mucosa cells before and after exposure to bitewing and panoramic radiographs, the following conclusions were drawn:

- Bitewing radiography causes a threefold increase in MN frequency post exposure (group 1B) compared with that before exposure (group 1A), with a statistically significant *P* value (*P*<0.001).
- Digital panoramic radiographs cause a twofold increase in MN frequency post exposure (group 2B) compared with that before exposure (group 2A), with a statistically significant *P* value (*P*<0.001).
- The study emphasized the fact that frequency of micronuclei increases post exposure to both bitewing and digital dental panoramic radiographs in children. Thus, increase in radiation exposure time and less scatted radiation increase the occurrence of micronuclei frequency in children.
- Bitewing and panoramic radiography should be advised only when it is necessary because it cannot be considered a risk-free procedure.

## Discussion

It is important to realize that children are often subjected to radiographs as part of dental treatment. They are thus at higher risk from radiation exposure compared with adults as the tissues of children are still in the development stage and are more sensitive to radiation. Children have a longer life span but are more susceptible to tumors. The effects of radiation are cumulative. Because of their smaller stature they are closer to the central X-ray beam.^9^ Hence, the present study aimed to evaluate the genotoxic effects of bitewing and digital dental panoramic radiography in children using BMCyt assay.

Detection of developmental conditions such as missing teeth, supernumerary teeth, ectopic eruption, delayed root resorption of primary teeth, deflected eruptive paths of permanent teeth, and caries activity is important for the optimal development of a child’s dentition. Radiographic examination involving bitewing and panoramic radiographs is an important tool for the proper diagnosis and monitoring of the above-mentioned conditions in children during the mixed dentition stage. Pediatric dentists routinely recommend either bitewing or panoramic radiographs, or both, as appropriate, for the diagnosis of various conditions in children.^10^

Bitewing films are used as ‘cavity detecting’ films to detect incipient interproximal caries, determine pulp chamber configuration and depth of carious lesions, record the width of spaces created by premature loss of primary teeth, evaluate the presence or absence of premolar crowns, and analyze the relation of the occlusal plane to possible tooth ankylosis.^10^

**Figure 1:**
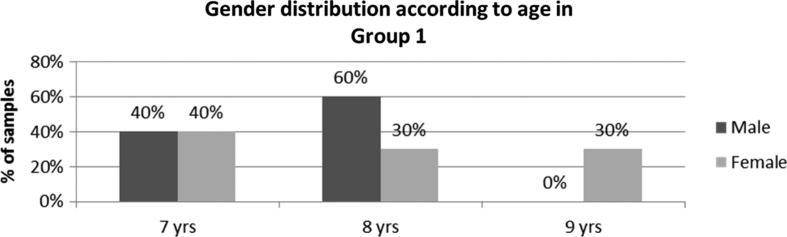
Distribution of male and female patients in group 1 (bitewing radiography) according to age in the study.

In our study, we have selected children who were diagnosed with moderate caries activity for bitewing radiographs (Table 1; Figure 1). Nowak *et al.*^11^ suggested that the child with high or moderate risk to dental caries should have bitewing radiographs taken as soon as possible, as the posterior primary teeth are in proximal contact and for this the age of the patient is not an important variable. If proximal caries is detected, follow-up radiographs are indicated semiannually until the child is caries free.^11^

**Figure 2:**
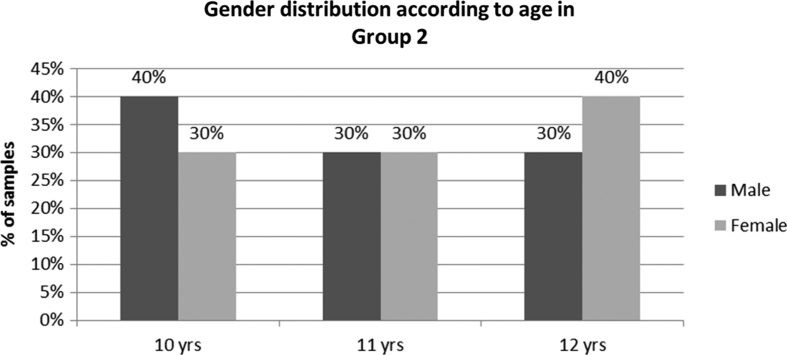
Distribution of male and female patients in group 2 (panoramic radiography) according to age in the study.

Panoramic radiographs are used to diagnose missing teeth, supernumery teeth, gross pathoses, ectopic eruption, delayed root resorption of primary teeth, and deflected eruptive paths of permanent teeth, evaluate skeletal and dental growth, and assess the development of dentition and malocclusion. In our study, we have included those children who were advised for panoramic radiographs (Table 2; Figure 2) for one of the above-mentioned conditions and who would seek orthodontic treatment. Various authors recommend that, regardless of risk level, all pediatric patients receive two panoramic radiographs, one at the early mixed dentition stage and one at the late mixed dentition stage, to detect undetected pathological conditions with the advantage of reduced dose and cost and imaging of a larger area.^10^

In our study, the genotoxic effects of the exposed dental radiation were evaluated using the BMCyt assay, which is a simple, noninvasive, cost-effective procedure for viewing a large number of exfoliated buccal mucosal cells.

Exfoliated buccal mucosal cells were collected to evaluate the MN frequency from radiation exposure before and after subjecting children to bitewing and digital dental panoramic radiography. An increase in MN frequency in exfoliated cells was observed as a result of radiotherapy. This was in relation to other studies,^12–16^ but to our knowledge this is the first study to evaluate the MN frequency from bitewing radiographs in children aged 6–9 years and from digital dental panoramic radiographs in children aged 10–12 years.

Exfoliated buccal mucosal cells have been used noninvasively to successfully show the genotoxic effects of lifestyle factors, medical treatments such as radiotherapy, as well as occupational exposure leading to potentially mutagenic and carcinogenic effects. These effects were evaluated from the buccal cells that turn over every 7–21 days, as it is theoretically possible to observe the genotoxic effects of an acute exposure only after 7–21 days.^17^

In our study, postexposure MN was evaluated 10±2 days after bitewing and digital dental panoramic radiographs, as the buccal cells in the basal layer were exposed to radiation initially and it takes 7–21 days to migrate to the superficial surface of the oral cavity.

According to Moore *et al.*, the BMCyt system can detect a 16-fold increase in MN frequency in oral cancer patients after completion of treatment with photons. The buccal mucosa also has the potential to be utilized to identify inherited genomic instability such as Bloom’s syndrome.^18^

The BMCyt assay thus has been used to measure biomarkers of DNA damage (micronuclei and/or nuclear buds) and also the cytokinetic defects (binucleated cells), proliferative potential (basal cell frequency), and cell death (condensed chromatin, karyorrhexis pyknotic, and karyolytic cells).^17^

In our study, the frequencies of MN cells were evaluated on the basis of exposure to both bitewing and digital dental panoramic radiography, similar to a few other studies carried out previously. The comparison carried out was unique to our study as previous studies did not attempt to compare the effects of dental radiation from bitewing and panoramic radiograph, as evaluated by fluorescence microscopy, in children.

**Figure 3:**
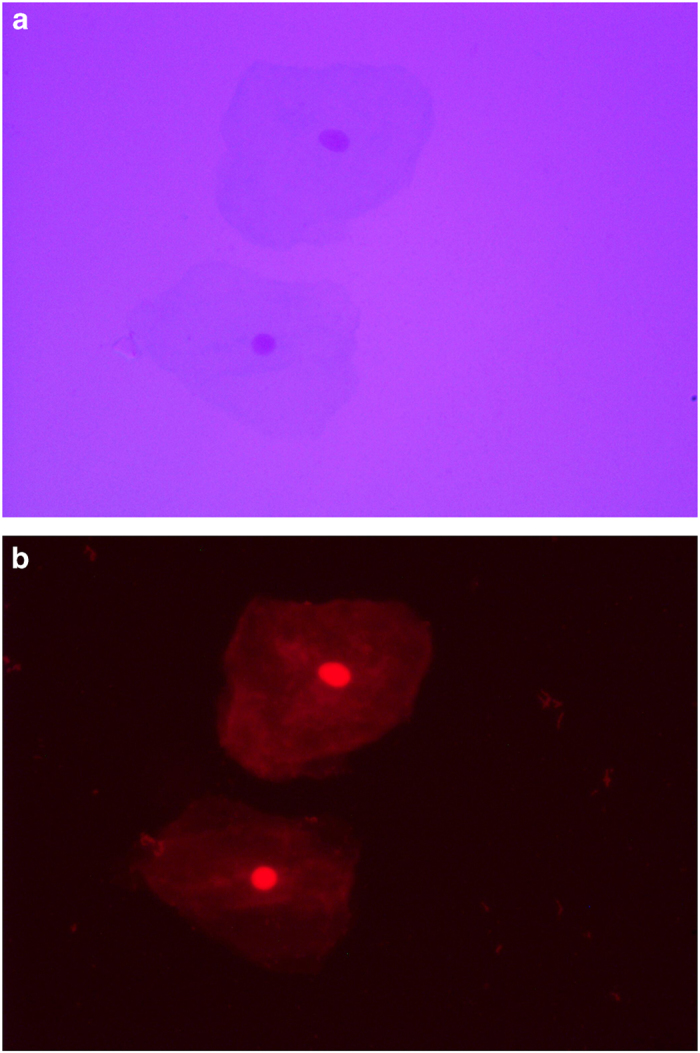
(**a**) Pre-exposed exfoliative buccal mucosal cells showing cytoplasm and nucleus under bright-field microscopy. (**b**) Pre-exposed exfoliative buccal mucosal cells showing cytoplasm and nucleus under fluorescence microscopy.

**Figure 4:**
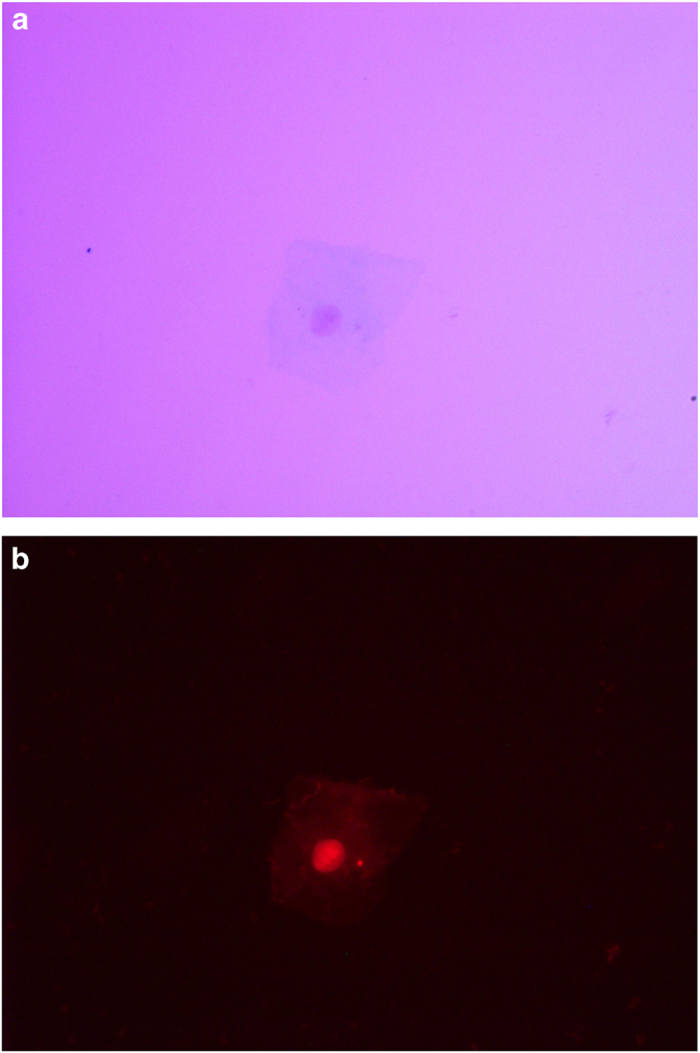
(**a**) Postexposed exfoliative buccal mucosal cells showing cytoplasm, nucleus, and micronuclei under bright-field microscopy. (**b**) Postexposed exfoliative buccal mucosal cells showing cytoplasm, nucleus and micronuclei under fluorescence microscopy.

In the present study, fluorescence microscopy was used to precisely identify and visualize the cell nuclei and MN using the Feulgen staining method, and evaluate false-positive MN count in bright field. Tools such as Romanowsky stains, propidium iodide, Hoechst 33258, or Acridine orange have resulted in a higher number of false positives, as they positively stain keratin bodies that are often mistaken for micronuclei; therefore, they are not reliable and hence were not used in this study. It is recommended to use Feulgen stain, which is a DNA-specific stain, and the permanent slides thus obtained can be viewed under both transmitted (Figures 3a, 4a) and fluorescent light (Figures 3b, 4b), as done in this study.^19^

Our study evaluated MN frequency before and after exposure to bitewing radiographs in children using the BMCyt assay. Bitewing radiographs showed a threefold increase in MN frequency post exposure (group 1 B) compared with that before exposure (group 1A; Table 3), with a statistically significant *P* value (*P*<0.001). The reason for the increase in MN count from bitewing radiation in children could be the direct point of focus of cone beam radiation on the buccal mucosal site of interest with comparatively decreased amount of scatted radiation; the targeted dosage of radiation in bitewing radiography was 70 Kvp.

In digital dental panoramic radiographs, the results showed a twofold increase in MN frequency post exposure (group 2B) compared with that before exposure (group 2A) (Table 4), with a statistically significant *P* value (*P*<0.001). The reason for the comparative decrease in MN count from digital dental panoramic radiation in children could be the divergent point of focus of cone beam radiation to the buccal mucosal site of interest as the tube head revolves around the jaw with the targeted dosage of radiation of 64 Kvp, which is less than that of bitewing radiography.

**Figure 5:**
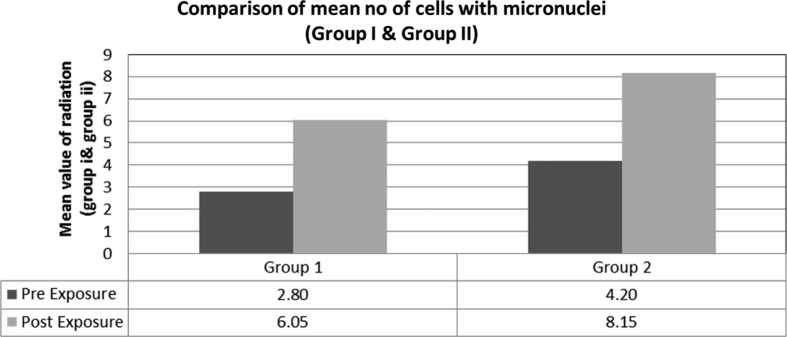
The comparison of micronuclei before and after exposure in both group 1 and group 2, with statistically significant difference.

Both groups showed statistically significant MN frequency before radiation (*Z* value −4.391; *P* value 0.001) and the relative increase in MN frequency post X-ray radiation in both groups was statistically significant (*Z* value −4.840; *P* value 0.001) (Table 5; Figure 5). Hence, in the present study, the micronuclei frequency was highly significantly elevated post exposure to bitewing and panoramic radiography when compared with that before exposure. These findings are similar to those of other studies conducted on panoramic radiographic exposure,^20,21^ which showed an increased frequency of micronuclei formation. A study by Lorenzoni *et al.* evaluated MN frequency after bitewing radiography along with other orthodontic radiographic procedures and found an increase in MN count. However, the difference was not statistically significant. In addition, other studies^14,22–24^ have shown a statistically nonsignificant difference in the frequency of micronuclei before and after exposure to panoramic radiography. The higher frequency of buccal epithelial micronuclei observed after X-ray exposure corroborates the data in the literature, in which X-ray radiation was reported to induce genetic damage that resulted in the increased formation of micronuclei in buccal epithelial cells as they are sensitive for detecting genotoxicants; the high radiation absorbed could be a reason for the positive result.^20^

It is important to note that micronuclei found in group 1 and group 2 before X-ray radiation may be due to diverse environmental and lifestyle factors such as a non-vegetarian diet, with no difference in gender distribution. This is commensurate with studies that have proved that diet and environmental factors have a direct influence on the increase in MN.^25^ In addition, exposure to X-ray radiation also significantly increases MN. Hence, the increase in micronuclei frequency found in the postexposure group could have been influenced by pre-exposure presence of micronuclei as well as postexposure radiation.

Even though the study was conducted under standard protocol, a certain amount of contamination was expected because of difficulty in efficiently standardizing the sample collection from young patients. Processing of the collected samples was time-consuming, and identification of MN proper was tedious, as we performed manual staining and a visual examination count. Although the study concluded statistically significant results, further studies with large sample sizes, which are epidemiological in nature and conducted under different clinical scenarios, with different age groups are required. Use of an automated machine for staining and counting cells will reduce time and bias. Advanced research using activated histone 2AX (Y-H2AX) and activated checkpoint kinase 2 (pChk2), which are DNA damage response molecules in irradiated cells,^26,27^ and fluorescence *in situ* hybridization analysis is also recommended, as it can serve as a sensitive indicator of low-dose radiation exposure in children.^28^

## Conclusion

The present *ex vivo* study was conducted to evaluate the extent of genetic changes on the basis of MN frequency in exfoliated buccal mucosa cells before and after exposure to bitewing and panoramic radiographs in children. It was concluded that the frequency of micronuclei increases post exposure in both bitewing and digital panoramic radiographs. Increased radiation exposure results in an increase in micronuclei frequency.^29–32^

## Figures and Tables

**Table 1:** Distribution of male and female patients according to age in study group 1 (bitewing radiography)

*Age (years)*	*Male*		*Female*	
	n	*%*	n	*%*
7	4	40	4	40
8	6	60	3	30
9	0	0	3	30
Total	10	100	10	100

**Table 2:** Distribution of male and female patients according to age in study group 2 (panoramic radiography)

*Age (years)*	*Male*		*Female*	
	n	*%*	n	*%*
10	4	40	3	30
11	3	30	3	30
12	3	30	4	40
Total	10	100	10	100

**Table 3:** Comparison of pre- and postexposure micronucleus cell count in group 1 (bitewing radiography)

*Exposure*	*Mean*	*s.d.*	*s.e. of mean*	*Mean difference*	*Z*	P-*value*
Pre-exposure to bitewing X-ray	2.80	0.70	0.16	−3.250	−3.940	<0.001^a^
Postexposure to bitewing X-ray	6.05	1.10	0.25			

The mean, s.d. and mean difference of pre- and post-micronucleus in group 1.

The increase in mean number of cells with micronucleus from pre-exposure to postexposure was found to be statistically significant (*P*<0.001).

a Significant difference.

**Table 4:** Comparison of pre- and postexposure micronucleus cell count within group 2 (panoramic radiography)

*Exposure*	*Mean*	*s.d.*	*s.e. of mean*	*Mean difference*	*Z*	P-*value*
Pre-exposure to panoramic X-ray	4.20	0.77	0.17	−3.950	−3.947	<0.001^a^
Postexposure to panoramic X-ray	8.15	0.81	0.18			

The mean s.d. and mean difference of pre- and post-micronucleus in group 2.

The increase in mean number of cells with micronucleus from pre-exposure to postexposure was found to be statistically significant (*P*<0.001).

a Significant difference.

**Table 5:** Comparison of mean number of cells with micronucleus between the two groups

*Cells*	*Group*	*Mean*	*s.d.*	*s.e. of mean*	*Mean difference*	*Z*	P-*Value*
Pre-exposure	Group 1	2.80	0.70	0.16	−1.400	−4.391	<0.001^a^
	Group 2	4.20	0.77	0.17			
Postexposure	Group 1	6.05	1.10	0.25	−2.100	−4.840	<0.001^a^
	Group 2	8.15	0.81	0.18			

Before exposure, a higher mean number of cells with micronucleus was recorded in group 2 compared with group 1, and the difference between them was statistically significant (*P*<0.001).

a Significant difference.

## References

[bib1] Johnson ON, Thomson EM. In: Essentials of dental radiography for dental assistants and hygienists, 8th edn. Inc: Upper Saddle River, NJ, USA, 2007.

[bib2] Kowitz AA, Loevy HT. Early periodical literature of dental radiology. Quintessence Int 2001; 32: 629–632. 11526891

[bib3] Brenner DJ, Doll R, Goodhead DT, Hall EJ, Land CE, Little JB et al. Cancer risks attributable to low doses of ionizing radiation: assessing what we really know. Proc Natl Acad Sci USA 2003; 100: 13761–13766. 14610281 10.1073/pnas.2235592100 PMC283495

[bib4] Cerqueira EM, Gomes-Filho IS, Trindade S, Lopes MA, Passos JS, Machado-Santelli GM. Genetic damage in exfoliated cells from oral mucosa of individuals exposed to X- rays during panoramic dental radiographies. Mutat Res 2004; 562: 111–117. 15279834 10.1016/j.mrgentox.2004.05.008

[bib5] Moore LE, Titenko-Holland N, Quintana PJ, Smith MT. Novel biomarkers of genetic damage in humans: Use of fluorescence in situ hybridization to detect aneuploidy and micronuclei in exfoliated cells. J Toxicol Environ Health 1993; 40: 349–357. 8230305 10.1080/15287399309531800

[bib6] Thomas P, Fenech M. Buccal micronucleus cytome assay. Method Mol Biol 2011; 682: 235–248. 10.1007/978-1-60327-409-8_17 21057932

[bib7] Vidya KB, Kalappanavar AN, Muniyappa M. Genotoxic effects of panoramic radiation by assessing the frequency of micronuclei formation in exfoliated buccal epithelium. Int J Res Med Sci 2014; 2: 541–544.

[bib8] Tolbert PE, Shy CM, Allen JW. Micronuclei and other nuclear anomalies in buccal smears: methods development. Mutat Res 1992; 271: 69–77. 1371831 10.1016/0165-1161(92)90033-i

[bib9] Myers DR. Dental radiology for children. Dent Clin North Am 1984; 28: 37. 6584345

[bib10] Mathewson RJ, Robert E. Fundamentals of Pediatric Dentistry 3rd edn. Primosch Quintessence Books, 1995.

[bib11] Nowak AJ. Radiation exposure in pediatric dentistry: an introduction. Pediatr Dent 1982; 3: 380.

[bib12] Torres-Bugarín O, Zavala-Cerna MG, Nava A, Flores-García A, Ramos-Ibarra ML. Potential uses, limitations, and basic procedures of micronuclei and nuclear abnormalities in buccal cells. Dis Markers 2014; 2014: 956835. 24778463 10.1155/2014/956835 PMC3932264

[bib13] Fucic A, Brunborg G, Lasan R, Jezek D, Knudsen LE, Merlo DF. Genomic damage in children accidentally exposed to ionizing radiation: a review of the literature. Mutat Res 2008; 658: 111–123. 18155954 10.1016/j.mrrev.2007.11.003

[bib14] Bonassi S, Coskun E, Ceppi M, Lando C, Bolognesi C, Burgaz S et al. The HUman MicroNucleus project on exfoliated buccal cells (HUMN(XL)):the role of life-style, host factors, occupational exposures, health status, and assay protocol. Mutat Res 2011; 728: 88–97. 21763453 10.1016/j.mrrev.2011.06.005

[bib15] Jois HS, Kale AD, Mohan Kumar KP. Micronucleus as potential biomarker of oral carcinogenesis. IJDA 2010; 2: 197–202.

[bib16] Popova L, Kishkilova D, Hadjidekova VB, Hristova RP, Atanasova P, Hadjidekova VV et al. Micronucleus test in buccal epithelium cells from patients subjected to panoramic radiography. Dentomaxillofac Radiol 2007; 3: 168–171. 10.1259/dmfr/29193561 17463102

[bib17] Thomas P, Holland N, Bolognesi C, Kirsch-Volders M, Bonassi S, Zeiger E et al. Buccal micronucleus cytome assay. Nat Protoc 2009; 4: 825–837. 19444240 10.1038/nprot.2009.53

[bib18] Rosin MP, German J. Evidence for chromosome instability in vivo in Bloom syndrome: increased numbers of micronuclei in exfoliated cells. Hum. Genet 1985; 71: 187–191. 4065890 10.1007/BF00284570

[bib19] Thomas P, Holland N, Bolognesi C, Volders KM, Bonassi S, Zeiger E et al. Buccal micronucleus cytome assay. Nat Protoc 2009; 4: 825–837. 19444240 10.1038/nprot.2009.53

[bib20] Vidya KB, Kalappanavar AN, Muniyappa M. Genotoxic effects of panoramic radiation by assessing the frequency of micronuclei formation in exfoliated buccal epithelium. Int J Res Med Sci 2014; 2: 541–544.

[bib21] Sheikh S, Pallagatti S, Grewal H, Kalucha A, Kaur H. Genotoxicity of digital panoramic radiography on oral epithelial tissues. Quintessence Int 2012; 43: 719–725. 23034425

[bib22] Angelieri F, de Oliveira GR, Sannomiya EK, Ribeiro DA. DNA damage and cellular death in oral mucosa cells of children who have undergone panoramic dental radiography. Pediatr Radiol 2007; 37: 561–565. 17453188 10.1007/s00247-007-0478-1

[bib23] da Silva AE, Rados PV, da Silva Lauxen I, Gedoz L, Villarinho EA, Fontanella V. Nuclear changes in tongue epithelial cells following panoramic radiography. Mutat Res 2007; 632: 121–125. 17574905 10.1016/j.mrgentox.2007.05.003

[bib24] Angelieri F, de Cássia Gonçalves Moleirinho T, Carlin V, Oshima CT, Ribeiro DA. Biomonitoring of oral epithelial cells in smokers and non-smokers submitted to panoramic X-ray: comparison between buccal mucosa and lateral border of the tongue. Clin Oral Investig 2010; 14: 669–674. 10.1007/s00784-009-0345-6 19798520

[bib25] Veerachari U, Venkatesh S, Yadav A, Narayanappa R. Biomonitoring genetic instability in normal healthy population using a simplecytogenetic marker—micronucleus test. Internat Multidiscipl Res. J 2011; 1: 01–09.

[bib26] Madhavan R, Kumaraswamy M, Kailasam S, Kumar SM. Genetic Damage in Exfoliated cells from oral Mucosa of individuals exposed to X-rays after Panoramic Radiograph:Across-sectional study. J Indian Aca Oral Med Radiol 2012; 24: 102–105.

[bib27] Yoon AJ, Shen J, Wu Hc, Angelopoulos C, Singer SR, Chen R et al. Expression of activated checkpoint kinase 2 and histone 2AXin exfoliative oral cells after exposure to ionizing radiation. Radiat Res 2009; 171: 771–775. 19580484 10.1667/RR1560.1 PMC3575577

[bib28] Decordier I, Mateuca R, Kirsch-Volders M. Micronucleus assay and labeling of centromeres with FISH technique. Methods Mol Biol 2011; 691: 115–136. 20972750 10.1007/978-1-60761-849-2_7

[bib29] Lorenzoni DC, Cuzzuol Fracalossi AC, Carlin V, Araki Ribeiro D, Sant Anna EF. Cytogenetic biomonitoring in children submitting to a complete set of radiographs for orthodontic planning. Angle Orthod 2012; 82: 585–590. 22149661 10.2319/072311-468.1 PMC8845552

[bib30] White SC, Pharoah MJ. Oral Radiology: Principles and Interpreatation 6th edn. Mosby: St Louis, MO, USA, 2009.

[bib31] Wojda A, Witt M. Manifestations of aging at the cytogenetic level. J Appl Genet 2003; 44: 383–399. 12923314

[bib32] Ishikawa H, Tian Y, Yamauchi T. Hitoshi Ishikawa, influence of gender, age and lifestyle factors on micronuclei frequency in healthy Japanese population. J Occup Health 2003; 45: 179–181. 14646294 10.1539/joh.45.179

